# Virtual fragment screening for novel inhibitors of 6-phosphogluconate dehydrogenase

**DOI:** 10.1016/j.bmc.2010.05.077

**Published:** 2010-07-15

**Authors:** Gian Filippo Ruda, Gordon Campbell, Vincent P. Alibu, Michael P. Barrett, Ruth Brenk, Ian H. Gilbert

**Affiliations:** a Biological Chemistry and Drug Discovery, College of Life Sciences, University of Dundee, Sir James Black Centre, Dundee DD1 5EH, UK; b Infection and Immunity and Wellcome Trust Centre for Molecular Parasitology, Glasgow Biomedical Research Centre, University of Glasgow, Glasgow G12 8TA, UK

**Keywords:** Virtual fragment screening, *Trypanosoma brucei*, 6-Phosphogluconate dehydrogenase

## Abstract

The enzyme 6-phosphogluconate dehydrogenase is a potential drug target for the parasitic protozoan *Trypanosoma brucei*, the causative organism of human African trypanosomiasis. This enzyme has a polar active site to accommodate the phosphate, hydroxyl and carboxylate groups of the substrate, 6-phosphogluconate. A virtual fragment screen was undertaken of the enzyme to discover starting points for the development of inhibitors which are likely to have appropriate physicochemical properties for an orally bioavailable compound. A virtual screening library was developed, consisting of compounds with functional groups that could mimic the phosphate group of the substrate, but which have a higher p*K*_a_. Following docking, hits were clustered and appropriate compounds purchased and assayed against the enzyme. Three fragments were identified that had IC_50_ values in the low micromolar range and good ligand efficiencies. Based on these initial hits, analogues were procured and further active compounds were identified. Some of the fragments identified represent potential starting points for a medicinal chemistry programme to develop potent drug-like inhibitors of the enzyme.

## Graphical abstract

We report the identification of novel inhibitors of *Trypanosoma brucei* 6PGDH enzyme by virtual fragment screening.

## 1 Introduction

Human African trypanosomiasis (HAT or sleeping sickness) is one of the most widespread and lethal tropical diseases on the African continent.^1,2^ The causative organisms are the parasites *Trypanosoma brucei rhodesiense* and *T. b. gambiense.* This insect-transmitted infectious disease puts 60 million Africans at risk of death every year. Current treatments, however, are unsatisfactory due to toxicity, increasing treatment failures and the need for parenteral administration, which is difficult in a rural setting in Africa.

**Figure 1:**
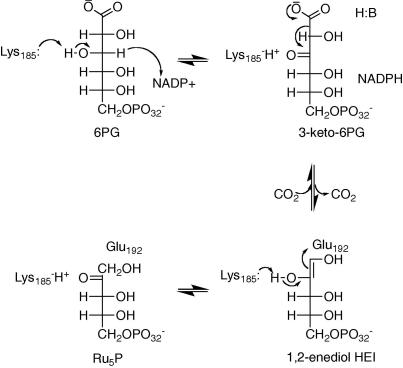
Catalytic mechanism of 6PGDH enzyme.

We have previously reported that the enzyme, 6-phosphogluconate dehydrogenase (6PGDH, EC 1.1.1.44), the third enzyme of the pentose phosphate pathway, is a potential drug target for HAT.^3–6^ 6PGDH catalyses the NADP-dependent oxidative decarboxylation of 6-phosphogluconate (6PG) to ribulose 5-phosphate (Ru5P). The catalytic mechanism consists of an acid–base catalysis, which proceeds with formation of a 3-keto-phosphogluconate, followed by decarboxylation to the 1,2-enediol high energy intermediate (HEI) and tautomerisation to the final product (Fig. 1). The two main residues acting as general base and general acid (Glu192 and Lys185, human numbering) are strictly conserved in all species. Site directed mutagenesis and crystallographic evidence has proved the essentiality of these residues for enzyme activity.^7–10^

Expression of *T. brucei* 6PGDH appears to be essential for viability of *T. brucei*.^11^ Inhibition of the enzyme diminishes the cellular pool of NADPH making the parasite more vulnerable to oxidative stress and increases the levels of 6-phosphogluconate (6PG). 6PG is known to be a potent inhibitor of glycolysis,^12^ and as the bloodstream form of *T. brucei* relies exclusively on glycolysis as source of energy, the parasite is very sensitive to disruption of this pathway. Interestingly, however, 6PGDH depleted trypanosomes are still susceptible to death when grown using fructose which should bypass the lethal feedback loop between glycolysis and 6PG.

**Figure 2:**
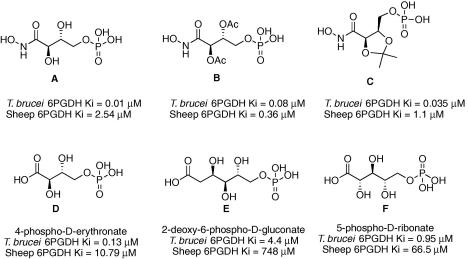
*T. brucei* 6PGDH inhibitors reported previously.^5,14^

We have characterised several *T. brucei* 6PGDH inhibitors^11^ and others are reported in the literature^13,14^ (Fig. 2). Most of these inhibitors are phosphorylated carboxylic acids derived from aldose sugars with poor drug-like properties. The three most potent and selective compounds are the hydroxamate analogues of the proposed transition state intermediate (compounds **A**–**C**, Fig. 2).^5^ Despite their potency (*K*_i_ values are in the nanomolar range) and their selectivity toward the parasite enzyme compared to the mammalian orthologue, these compounds do not have trypanocidal activity. They are analogues of 4-phospho-d-erythronate, with a sugar-like backbone, a carboxylic moiety and a phosphate group. Compounds of this type are predicted to have poor membrane permeability due to their charge and polarity.

Crystal structures of human, *T. brucei*, *Lactococcus lactis*, and *Geobacillus stearothermophilus* 6PGDH have been determined and deposited in the PDB.^7,15–20^ All residues that interact with the substrate are fully conserved between *Tb*6PGDH and *Ll*6PGDH. *Ll*6PGDH, in contrast to *Tb*6PGDH, was co-crystallised with substrate and inhibitors.

There are two methods in drug discovery, which exploit crystal structure information for hit discovery: protein structure-based virtual screening and fragment-based hit discovery.^21–24^ In protein-based virtual screening, constraints are derived from the crystal structure and used to filter in silico libraries for compounds fulfilling these constraints. In fragment-based design, libraries typically containing small molecules with a molecular weight of less than 300 Da, and less than three hydrogen-bond donors and six hydrogen-bond acceptors are screened using biophysical methods such as X-ray crystallography, nuclear magnetic resonance (NMR) or surface plasmon resonance (SPR). The resulting hits are often weak inhibitors with binding constants in the high micromolar to low millimolar range, but high ligand efficiencies. The next stage in this strategy is to optimise these fragments to more potent compounds, by adding substituents which make further binding interactions with the enzyme active site. This process can be facilitated when the binding modes of the hits and derivatives are determined crystallographically. It is possible to combine both strategies by restricting virtual screening to fragment-like libraries.^22,25–27^

In this paper, we report the virtual fragment screening of 6PGDH with the goal of finding fragments that are suitable starting points for a drug discovery programme. A potentially confounding issue with the active site of 6PGDH is that it contains a large number of polar residues required for binding to the phosphate, carboxylic acid and hydroxyl groups of the substrate. A corollary of this is that effective inhibitors targeted against the substrate binding site are also likely to be polar and hence not very drug-like. We decided to use the structural information available on 6PGDH to find inhibitors that have a relatively low polarity and have the correct physicochemical properties to be orally bioavailable. A fragment-based approach is attractive, as it should allow us to build molecules into less polar regions of the active site, to derive more ‘drug-like’ inhibitors.

## 2 Results

The binding sites of *Ll*6PGDH and *Tb*6PGDH are highly conserved but only *Ll*6PGDH has been co-crystallised with cofactor and substrate or inhibitor.^15^ Therefore, we used the latter crystal structure of *Ll*6PGDH in complex with NADP^+^ and PEX (compound A, Fig. 2) as a template structure for virtual screening.

**Figure 3:**
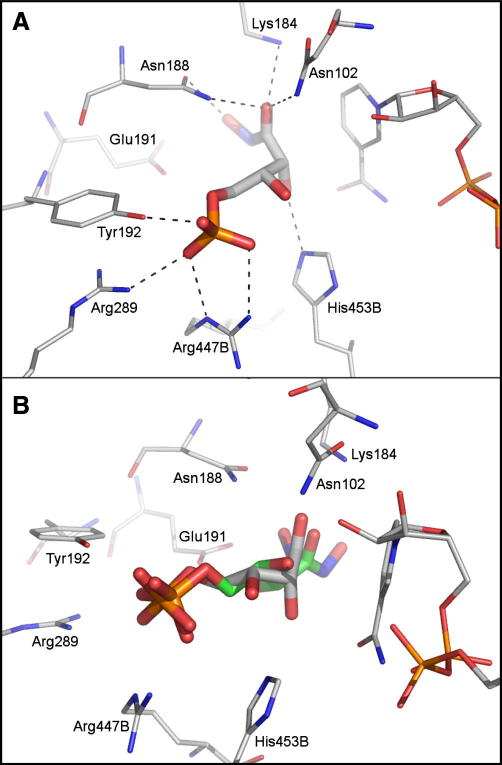
(A) Ligand PEX in the active site of *L. lactis* 6PGDH. Putative hydrogen bonds are indicated by dashed lines. (B) Superposition of the ligand PEX (green carbon atoms) with the binding mode of the same ligand predicted by the docking calculations (grey carbon atoms). The RMSD between both posed is 1.16 Å.

The 6PGDH-binding site contains a cluster of positively charged residues, which accommodates the phosphate group of the substrate (Fig. 3A). This part of the active site is deeply buried in the enzyme active site and not readily accessible by bulk solvent. We therefore assumed that by targeting this area potent inhibition can be achieved. Consequently, the docking programme used in this study (DOCK 3.5^28,29^) was biased to address this part of the binding site by only placing spheres for ligand matching close to the cluster of positively charged residues. This set-up was validated using a set of 36 *Tb*6PGDH inhibitors^5,6,30^ (33 moderate inhibitors plus the three potent compounds **A**–**C**). Re-docking PEX into the *Ll*6PGDH resulted in a docking pose close to the crystallographically determined binding mode (RMSD 1.16 Å) with the phosphate group interacting with the two arginine residues (447 and 289) and the Tyr192 hydroxyl group (Fig. 3B). The docking programme was also able to generate plausible binding modes for most inhibitors of the test-set; the phosphate groups of the compounds were placed into the same area where the phosphate group of PEX binds, albeit no crystal structures were available to compare the predicted binding poses.

The goal of this study was then to identify new scaffolds for the potential development of inhibitors of *T. brucei* 6PGDH by virtual fragment screening. These fragments could potentially be elaborated to pick up further binding interactions with the enzyme active site, and hence increase the potency of inhibition. One key requirement, for compounds likely to show oral bioavailability, was to replace the phosphate group found in both the substrate and known inhibitors (Fig. 2) with functional groups that are less polar and ‘less ionised’ at physiological pH. The phosphate replacement should still be able to bind strongly to the cluster of positively charged amino acids known to bind to the phosphate. The available chemicals and screening compounds directories (ACD–SCD) were consequently filtered for compounds containing any of the following functionalities that may be able to mimic the phosphate: phosphonate, sulfonate, sulfonic acid, sulfonamide, carboxylic acid, and tetrazole. In addition, the compounds were required to have a molecular weight of less than 320 Da. Applying these filters resulted in a library containing approximately 64,000 compounds.

The filtered sub-set was docked into the *Ll*6PGDH-binding site using DOCK 3.5.54 wherein the ligands were forced to place their negatively charged functional group into the positively charged area of the binding site. Out of the 64,000 compounds in the filtered set, binding modes for 5836 compounds were generated. For the remaining compounds the docking programme was not able to generate a docking pose due to steric clashes. The 5836 hits were subsequently clustered using Daylight fingerprints and ranked within each cluster according to their docking score, resulting in around 500 clusters and approximately 400 singletons. High scoring compounds were visually inspected together with the compounds belonging to the same cluster and 71 promising compounds were identified for purchase. Criteria for visual inspection were quality of hydrogen bonds formed with the amino acids of the positively charged cluster and diversity of chemical scaffolds.

**Figure 4:**
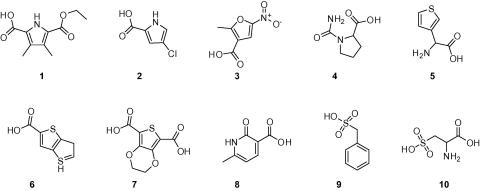
Hits identified in the first screening with percentage of inhibition higher than 80% at 200 μM.

**Figure 5:**
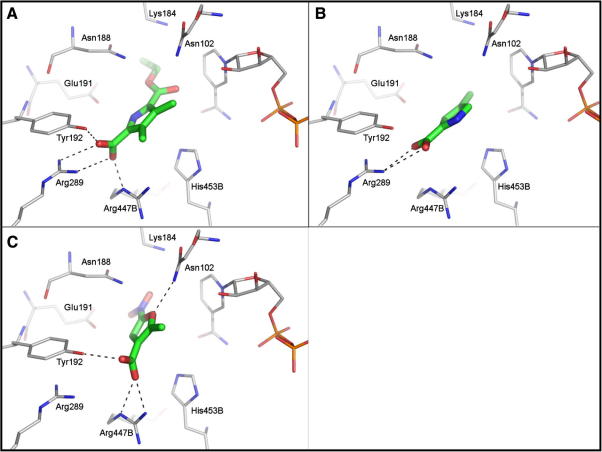
Docking poses for compound **1** (A), compound **2** (B) and compound **3** (C) in the active site of *L. lactis*.

**Figure 6:**
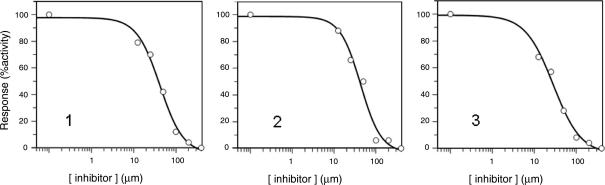
IC_50_ curves for the compounds **1**–**3**.

The selected compounds were evaluated in a biological assay using the recombinant *Tb*6PGDH. Initially, percent inhibition of the compounds were determined at 200 μM. Compounds which showed a percentage of inhibition equal or greater than 80% were re-screened at 50 μM (Fig. 5). Finally, IC_50_ determinations were carried out on compounds giving >50% inhibition at 50 μM. Three compounds gave IC_50_ values in the low micromolar range (Table 1) and were considered of interest for further study. We were unable to obtain IC_50_ values with the remaining compounds (**4**–**10**) in Figure 4 and assume that these were false positives in the initial screen or that compounds were labile over the storage period. The ligands **1**–**3** contain only between 11 and 14 non-hydrogen atoms and have therefore high ligand efficiencies^22,31^ of 0.4–0.7 [(kcal/mol)/heavy atom], their IC_50_ curves are also reported in Figure 6. In the computationally generated binding modes the carboxylate groups of the ligands interact with the positively charged area in the binding site which previously had been observed to accommodate the phosphate group of the substrate of substrate-like inhibitors^15^ (Fig. 5A–C).

To further validate the initial hits the ACD–SCD database was searched for commercially available analogues of the hits in Figure 4. An additional 53 compounds were purchased (together with hits from the first screening) and tested for enzyme inhibition. For the most active compounds IC_50_ values were determined (compounds **11**–**18**, Table 1). The compounds **15**–**18,** have Hill slopes that are much higher than expected for competitive inhibitors and, most likely, are promiscuous inhibitors.^32,33^ Compounds **4**, **5** and **10**, did not confirm the previous reported activity, indicating that perhaps they were false positives in the first screening. The most potent and interesting hits were compounds **1**–**3**, for which the IC_50_ curves are reported in Figure 6 (identified in the first screening and re-confirmed in the second round) and **11**–**13** with IC_50_ values between 40 and 80 μM, all with ligand efficiency higher than 0.3 (such a ligand efficiency is required for compound of molecular weight 500 Da to have an IC_50_ value of 10 nM).

## 3 Discussion

6-Phosphogluconate dehydrogenase is a potential drug target for human African trypanosomiasis. The enzyme has been demonstrated to be essential by RNAi studies,^11^ and inhibitors have been discovered that inhibit the enzyme potently and with good selectivity compared to a mammalian (sheep liver) 6PGDH (Fig. 2).^5,14^ In order to accommodate the polar substrate, the active site of 6PGDH is correspondingly polar (Fig. 3A). The inhibitors that we have previously developed^5^ are based around the substrate and are polar themselves. Whilst they are potent inhibitors of the enzyme, they lack drug-like properties, in particular cellular permeability. We have published a prodrug approach for selective delivery of these compounds which demonstrates how even highly polar compounds have potential for development as drugs.^4^ An alternative strategy, for a discovery process, is to search for new chemical starting points that are inherently less polar. In this paper we report an approach to do this by selecting an in silico library of fragments from commercially available compounds and then carrying out a virtual screen of the library. From the hits identified by the computational work, compounds were purchased and tested for inhibition of the enzyme. Information from the first round of testing was used to guide a second round of compound purchase. Future work should now focus on building upon the fragment scaffolds in order to increase their potency as inhibitors, taking advantage of other interactions within the enzyme’s active site, and ultimately to develop them as compounds with better drug-like properties.

Virtual screening of the fragment library and subsequent purchase and testing of analogues identified several ligands with IC_50_ values in the low micromolar range, and with high ligand efficiencies (Table 1). Except for compound **4**, all of the ligands have a carboxylate group as a replacement for the phosphate; in the generated binding modes the carboxylate mimics the phosphate group (Fig. 5). Examples of a carboxylate group mimicking phosphate interactions have already been found in the past for various cases, including protein tyrosine phosphatase 1B (PTP1B).^34^ Compared to the phosphate group a carboxylic group has the advantage that the p*K*_a_ is much higher (∼2 for phosphate vs ∼5 for carboxylate) and the discovered hits have therefore a much better chance of being membrane permeable.

Interestingly other functional groups selected for the screening (tetrazole and sulfonamides) did not produce good hits. This could probably be explained considering the higher chemical diversity present among the carboxylic acids, which were higher in number compared to the tetrazoles and sulfonamides in the starting database.

Solubility, partition coefficients, TPSA and human intestinal absorption (HIA) were calculated using the software Stardrop (Optibrium Ltd, Cambridge, UK) and are reported in Table 1. Comparison of these predictions with those for the known inhibitors, reported in Table 2, clearly shows how the new scaffolds constitute an improvement in the drug-likeness of the molecules. The partition coefficients are higher for compounds **1–18** than the corresponding values for the inhibitors **A**–**C**, indicating a higher likelihood of passive diffusion. Similarly, in general, the TPSA values are more reasonable for an orally bioavailable compound. Human intestinal absorption (HIA) is also estimated, as a predictor for oral bioavailability. This is a positive value (predicted absorption is ⩾30%) for all the hits in Table 1 whereas compounds **B** and **C** have negative signs, again indicating a low likelihood of oral bioavailability.

From the chemistry point of view the aldose sugar moiety could be replaced with some five-membered heterocycles (compounds **1–3**) or fused five/six-membered rings (compounds **12** and **13**) indicating that the binding pocket is large enough to accommodate these types of structures. This finding is also supported by the fact that 6-aminonicotinamide^35^ was reported as a potential inhibitor of 6PGDH, although this compound might bind to the cofactor binding site. These new heterocyclic scaffolds constitute valuable starting points for further optimisation and give the opportunity to explore new, tractable, chemistry routes. By building towards the less polar cofactor binding site and exploiting additional interactions with the enzyme it should be possible to improve their affinities. The generated docking poses can serve as guidelines for structure-based design to support this process. However, during docking the ligands were forced to place their charged group into the area with which the phosphate groups of substrate and substrate-like inhibitors interact. Unrestrained docking of the hits discovered here into the 6PG binding site indicated that alternative interactions of the carboxylate group with the area in which the carboxylate group of the substrate binds could also be possible. Attempts are therefore underway to determine crystallographically the binding modes of the discovered hits.

## 4 Conclusions

Virtual screening successfully identified new scaffolds, which can bind to the *Tb*6PGDH active site. The 6PGDH active site is polar, yet the compounds that we have discovered include some less polar molecules that have good ligand efficiencies (>0.3 kcal/mol/(HAC)) and promising physicochemical and predicted ADME properties. Some of the hits are chemically tractable and have the potential to be elaborated further to drug-like molecules.

## 5 Methods

### 5.1 Compound selection and quality control

The ACD–SC library was screened for molecules which have a molecular weight of less than 320 Da and which contain at least one of the following negatively charged functional groups: phosphonate, sulfonate, sulfonic acid, sulfonamide, carboxylic acid, bicarboxylic acid or tetrazole.

The search was carried out through the DiscoveryGate webservice (Symyx Technologies Inc.) on the ACD–SC database. The second selection of compounds was carried out by using a similarity index of 80 in respect to the structures of the hits identified in the screening.

Once purchased the compounds were tested for quality control by LC–MS on a Bruker Microtof spectrometer. The compounds eluted through a Waters Xbridge column (5 × 50 mm), with a gradient of 5–95% ACN/water +0.1% ammonia and they showed a purity >95%.

### 5.2 Computational methods

DOCK 3.5.54^28,29^ was used to dock small molecules into the active site of *Ll*6PGDH. In short, DOCK 3.5.54 uses a hierarchical database format to dock conformational ensembles of small molecules into protein sites.^27^ The conformations of a given molecule are pre-calculated and superimposed on a rigid common fragment, which is usually a ring system. The ligand conformers are then docked together, as an ensemble, into a receptor binding site by matching the atoms of the common fragment to receptor spheres. In the current study, we wanted to generate only ligand poses in which the functional groups intended to replace the phosphate group were placed in the phosphate binding site. To achieve this, the following changes were made to our standard receptor and database preparation procedures published earlier:^25^ (1) Instead of superimposing the small molecule ensembles on the ring systems the phosphate isostere groups were used as common fragments for compound alignment and matching. (2) Spheres used for receptor matching positions for docking were only placed in the binding site in the area that was occupied by phosphate group of the inhibitor.

The crystal structure of *Ll*6PGDH in complex with cofactor and PEX (2IZ0) was chosen as template structure.^15^ The protonation states of the two catalytically important residues Lys184 and Glu191 changes following the reaction coordinate. Here, we only considered the protonation states which are required to bind the substrate, in particular, Lys184 was considered to be positively charged and Glu191 to be neutral.

To facilitate visual inspection all compounds for a binding mode was generated were clustered using the Jarvis–Patrick algorithm of the Daylight clustering package (Daylight, Aliso Viejo, USA) whereas compounds had to have at least 10 of 16 neighbours in common in order to be allowed to belong the same cluster and compounds for which the nearest neighbour had a Tanimoto coefficient of less than 0.8 were classified as singletons.

### 5.3 Assay

The *T. brucei* 6PGDH expressed in *E. coli* was purified as described.^36^ Inhibition studies involved a reaction in 50 mM triethanolamine pH 7.0, 2 mM MgCl_2_. NADPH and 6PG were each at 20 μM. Total reaction volume was 1 ml. The reaction was followed in a Perkin Elmer UV–vis spectrophotometer. Compounds were dissolved in DMSO and initially added at 200 μM, then 50 μM. Any compound giving more than 50% inhibition at 50 μM was used to determine IC_50_ values over a range of substrates (doubling dilutions from 200 μM).

## Figures and Tables

**Table 1:** IC_50_ values, Hill slopes, ligand efficiency (−RTLn(IC_50_)/HAC)^31^ and predicted solubility, log *P*, log *D*, human intestinal absorption (HIA) and TPSA using StarDrop

Compd	Structure	IC_50_ (μM)	Hill slope	Ligand efficiency [(kcal/mol)/HAC]	Log *S*	Log *P*	Log *D*	HIA	TPSA
**1**		45 ± 11	1.6	0.40	3.3	1.7	−1.1	+	79
**2**		43 ± 9	1.8	0.66	4.3	1.4	−1.6	+	53
**3**		28 ± 6	1.5	0.52	4.2	1.1	−1.4	+	96
**11**		73 ± 9	1.4	0.33	2.4	0.7	0.7	+	93
**12**		51 ± 3	1.7	0.42	3.2	1.2	−1.1	+	68
**13**		42 ± 7	1.1	0.54	3.4	1.5	−1.2	+	53
**14**		114 ± 27	1.4	0.27	2.6	2.1	−1.1	+	106
**15**		56 ± 6	5.2	0.36	3.4	1.3	−3.2	+	134
**16**		53 ± 4	4.8	0.36	2.2	−0.2	−0.2	+	78
**17**		5.8 ± 0.6	2.0	0.48	3.4	0.9	−1.9	+	120
**18**		42 ± 3.7	2.1	0.60	3.9	2.0	−1.1	+	53

HAC: heavy atom count. StarDrop: (www.optibrium.com).

**Table 2:** StarDrop predictions for the known inhibitors **A**−**C**

	Log *S*	Log *P*	Log *D*	HIA category	MW	HBD	HBA	TPSA
**A**	5.4	−0.9	−2.2	+	315	4	11	169
**B**	5.6	−0.2	−2.5	−	271	4	9	135
**C**	6.2	−1.8	−3.9	−	231	6	9	157

StarDrop: (www.optibrium.com).
